# Tissue-specific expression of p73 and p63 isoforms in human tissues

**DOI:** 10.1038/s41419-021-04017-8

**Published:** 2021-07-27

**Authors:** Clayton B. Marshall, J. Scott Beeler, Brian D. Lehmann, Paula Gonzalez-Ericsson, Violeta Sanchez, Melinda E. Sanders, Kelli L. Boyd, Jennifer A. Pietenpol

**Affiliations:** 1 Department of Biochemistry, Vanderbilt University, Nashville, TN USA; 2 Department of Medicine, Vanderbilt University, Nashville, TN USA; 3 Vanderbilt-Ingram Cancer Center, Vanderbilt University Medical Center, Nashville, TN USA; 4 Department of Pathology, Microbiology and Immunology, Vanderbilt University, Nashville, TN USA; 5 Gilead Sciences Inc., Foster, CA USA

**Keywords:** RNA, Cellular imaging, Transcription

## Abstract

p73 and p63 are members of the p53 family that exhibit overlapping and distinct functions in development and homeostasis. The evaluation of p73 and p63 isoform expression across human tissue can provide greater insight to the functional interactions between family members. We determined the mRNA isoform expression patterns of *TP73* and *TP63* across a panel of 36 human tissues and protein expression within the highest-expressing tissues. *TP73* and *TP63* expression significantly correlated across tissues. In tissues with concurrent mRNA expression, nuclear co-expression of both proteins was observed in a majority of cells. Using GTEx data, we quantified p73 and p63 isoform expression in human tissue and identified that the α-isoforms of *TP73* and *TP63* were the predominant isoform expressed in nearly all tissues. Further, we identified a previously unreported p73 mRNA product encoded by exons 4 to 14. In sum, these data provide the most comprehensive tissue-specific atlas of p73 and p63 protein and mRNA expression patterns in human and murine samples, indicating coordinate expression of these transcription factors in the majority of tissues in which they are expressed.

## Introduction

p73 and p63 are members of the p53 family of sequence-specific transcription factors that directly regulate differentiation, cell cycle, proliferation, DNA repair, and apoptosis [1–5]. p73 and p63 bind similar consensus DNA binding sites [6, 7]; but diverge in their regulation of gene targets [8–11]. Further, p73 and p63 can heterodimerize to differentially co-regulate downstream target genes [12–15].

The functional and physical interactions between p73 and p63 lead to complicated, multi-faceted regulation of target genes. p73 is co-expressed with p63 in the basal cell populations of many epithelial tissues [16]. Previously, we discovered that 50% of basal epithelial cells in the trachea express p73 and that the tracheas from p73-deficient (p73−/−) mice have a 35% reduction of basal cells in addition to a loss of multiciliated epithelial cells [17]. p73 protein expression is a marker of the basal epidermal stem cell populations located near hair follicles in scale-like skin [18]. Further, our laboratory found that p73−/− mice have delayed epidermal wound healing attributable to regulation of genes involved in proliferation, DNA damage response, cellular junctions, and skin development [19]. These results collectively suggest a role for p73 in basal epithelial cells, and highlight the need to examine further the coordinate isoform expression of p73 and p63 across human tissues.

Both p73 and p63 genes have two promoters (P1 and P2) that give rise to isoforms with distinct transcriptional programs [1, 5, 9, 20–23]. Isoforms transcribed from P1 contain a full-length transactivation (TA) domain [1, 5], while isoforms transcribed from P2 have a truncated TA (∆N) domain [5, 20]. The *TP73* and *TP63* genes also undergo C-terminal alternative splicing, which generates several isoforms (termed α, β, γ, etc.) with different activity at target genes [1, 5, 24, 25]. p73 has also been reported to undergo N-terminal alternative splicing that removes exons from the TA domain (∆ex2 and ∆ex2/3) [26, 27].

Large-scale analysis of RNA-seq datasets have been previously performed for TP*63* isoforms [28]; however, there was an unmet need to complete a similar analysis for p73 and integrate its expression patterns with p63. Thus, we analyzed p73 expression using human tissue RNA-seq data from the GTEx (Genotype-Tissue Expression) Project [29, 30]. In parallel, we also analyzed p63 expression to determine if coordinate p73 and p63 expression occurred and compared the protein expression patterns between human and murine tissue. To overcome the challenges of quantifying p73 and p63 mRNA isoforms, we developed an algorithm to analyze exon junction-spanning reads and independently quantified N-terminal and C-terminal isoform expression of p73 and p63. We found that p73 and p63 expression correlated across tissues, with most tissues expressing both genes or neither. We analyzed RNA-seq data to determine the expression of p73 alternative promoter usage isoforms in epithelial tissues and provide evidence suggesting the existence of a novel p73 transcript encoded by exons 4 through 14. Our analyses are the most extensive to date on p73 and p63 protein and mRNA expression patterns within normal adult tissues from both humans and mice. We observe coordinate expression patterns of p73 and p63 in basal cell populations of epithelial tissues.

## Results

### *TP73* and *TP63* gene expression in human tissue

**Fig. 1:**
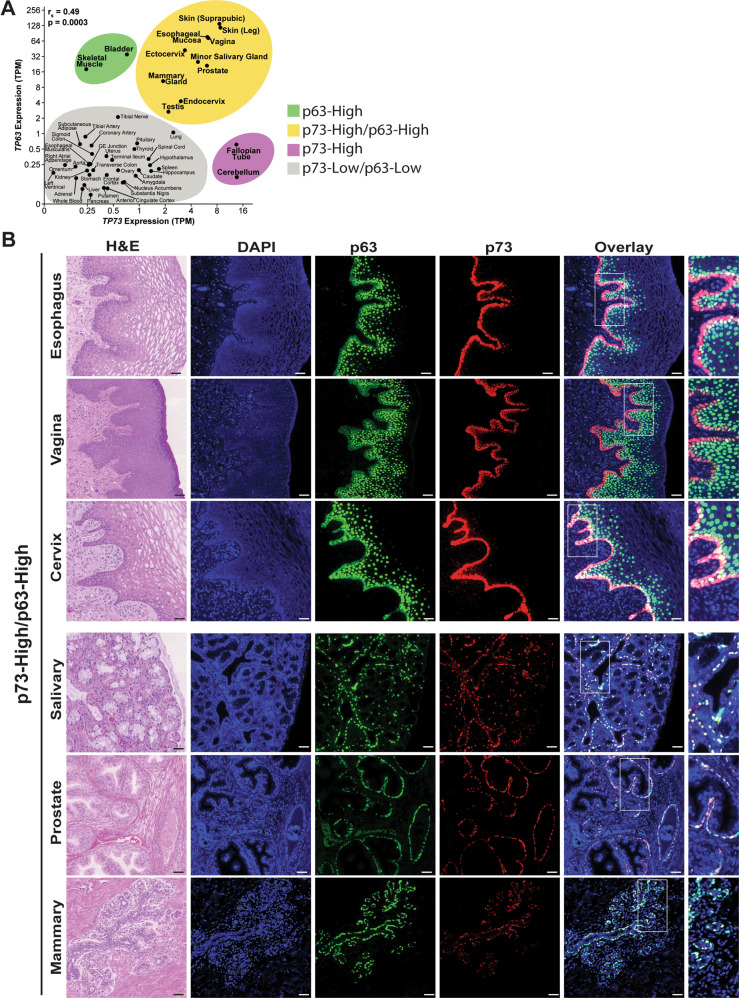
p73 and p63 Gene and protein expression in human tissue. **A** *TP73* versus *TP63* mRNA expression (units = TPM) for each human tissue type in the GTEx dataset. The mean expression (TPM + 0.1) for each tissue is plotted on a log_2_ scale. Correlation between *TP73* and *TP63* was quantified using Spearman’s rank correlation coefficient (*r*_s_). Tissues were divided into four groups based on the patterns of p73/p63 expression: p73-High/p63-High (yellow shading), p73-High (purple shading), p63-High (green shading), and p73-Low/p63-Low (gray shading). **B** Representative H&E and dual IF micrographs (serial sections) from the p73-High/p63-High group described in Part A. p73 (red), p63 (green), and DAPI stained nuclei (blue). For each normal tissue site, samples from three different humans were stained and analyzed to identify the representative area shown. Scale bars = 50 µm.

To determine the expression patterns of *TP73* and *TP63* in human tissue, we analyzed RNA-seq data from the GTEx Project [29, 30]. We observed a significant correlation (*r*_s_ = 0.49, *p* = 0.0003) between *TP73* and *TP63* expression across all tissues and found that *TP63* transcript was expressed at higher levels than TP73 in most tissues (Fig. 1A). In our previous work, we observed similar gene expression using RNA-seq data from the Human Protein Atlas [19]. We identified four major groupings of *TP73/TP63* expression across tissues. The majority of tissues expressed low-levels [<2 transcripts per million (TPM)] of both *TP73* and *TP63* (p73-Low/p63-Low) (Fig. 1A, gray shading). The second group of tissues expressed elevated levels (>2 TPM) of both *TP73* and *TP63* (p73-High/p63-High) (Fig. 1A, yellow shading). A small number of tissues preferentially expressed *TP73* (p73-High) (Fig. 1A, purple shading) or *TP63* (p63-High) (Fig. 1A, green shading).

### Atlas of p73 and p63 protein expression in human and murine tissue

Previous publications have examined the protein expression patterns of p73 and p63 in select murine and human tissues, including the mammary gland [31], ovary [8, 31, 32], fallopian tube [17], lower respiratory tract [17, 33, 34], testis [17, 35, 36], brain [20, 37–41], and skin [19, 25, 42]. In a comprehensive approach, we performed dual p73 and p63 immunofluorescence (IF) alongside serial sections stained with hematoxylin and eosin (H&E) to determine p73 and p63 protein expression patterns in human tissues (Fig. 1A).

Consistent with RNA expression in the p63-High group (Fig. 1A, green shading), we observed p63-positive staining in both skeletal muscle and bladder with little p73 expression in either human (Fig. S1A) and murine (Fig. S2A) tissue. Of note, the expression pattern in skeletal muscle is corroborated by a previous report that observed p63 staining localizes to sarcomere Z-bands [43].

Similarly, the p73-High tissues (Fig. 1A, purple shading) of the cerebellum and fallopian tube displayed p73 expression with minimal or no p63 protein (human: Fig. S1B, mouse: Fig. S2B). Of note, cerebellar Purkinje cells exhibited more cytoplasmic expression of p73, while the granular layer displayed more nuclear staining (Fig. S1B). In the majority of epithelial tissues (esophagus, vagina, cervix, prostate, and salivary gland) from the p73-High/p63-High group (Fig. 1A, yellow shading), we found that p73 primarily co-localized with p63 in a subset of basally-located epithelial cell nuclei (human: Fig. 1B, mouse: Fig. S2C). The tissues with the highest co-expression levels were squamous epithelial (esophagus, vagina, and cervix). In contrast, the exocrine tissues (salivary, prostate, and mammary) exhibited lower p73 and p63 expression (Fig. 1B). Previous work from our laboratory focused on the role of p73 in the murine dorsal back skin [19]. We expanded on this and performed dual p73 and p63 IF staining on other human (face, gluteus, and abdomen/groin) and murine (dorsal back, toe, ear, and tongue) epithelia, and observed co-expression of p73 in a subset of basally-localized p63-positive cells in all epithelium analyzed (Figs. S1C and S3A).

### Alternative C-terminal splicing of *TP73* and *TP63* in human tissue

p73 and p63 isoforms have differential transcriptional activity [1, 5, 24, 25, 44] and isoform-specific knockout mice display varying phenotypes [20, 32, 38, 45–52], consistent with isoform-dependent roles for p73 and p63 in tissue development and homeostasis. Quantification of full-length *TP73* and *TP63* isoform expression using standard methodologies is challenging due to long transcript lengths, low relative expression levels, isoform variation at both N-terminal and C-terminal ends, and the lack of isoform-specific antibodies. To overcome these challenges and determine p73 and p63 isoform expression in human tissue, we developed an algorithm to quantify the mRNA expression of p73 and p63 isoforms across tissue types by analyzing exon junction-spanning reads from RNA-seq data. We quantified p73 and p63 isoform expression for each sample in GTEx using our algorithm, summarizing the results by the mean of each N-terminal and C-terminal isoform per tissue.

**Fig. 2:**
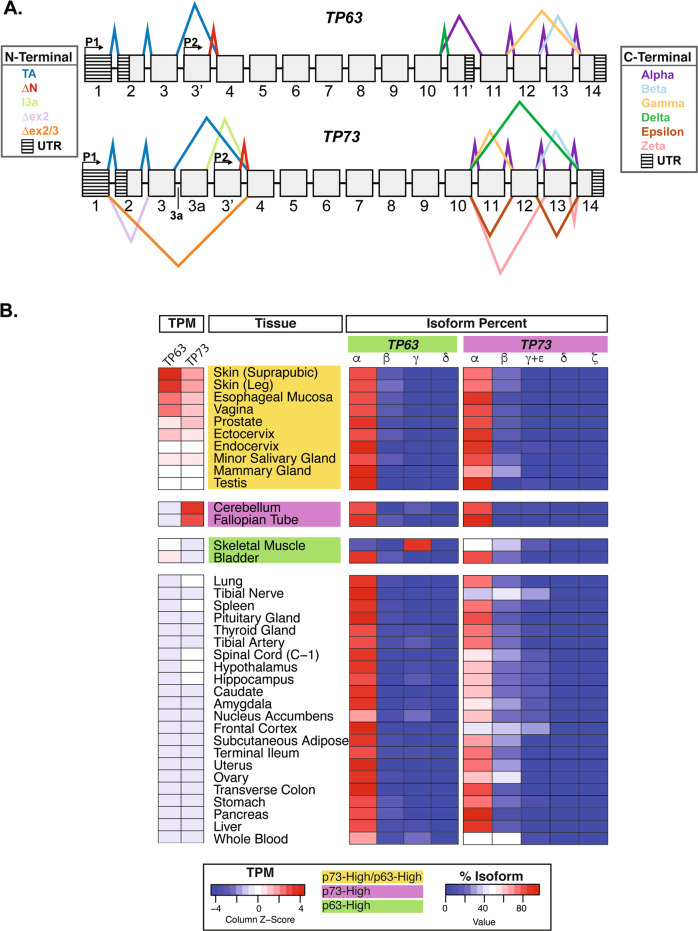
*TP73* and *TP63* mRNA isoform expression in human tissue. **A** Schematic representation of the exon structure and splicing of TP63 and TP73. Promoters (P1-TA and P2-∆N) are annotated with arrows. Untranslated regions (UTR) are annotated with horizontal stripes. Splicing events are annotated with colored lines, and the key to the line colors is located to the right of the schematic. **B** Heatmap of RNA-seq expression (TPM) for *TP63* and *TP73* in human tissue types from the GTEx dataset (left-sided heatmap) with the corresponding relative percentage of C-terminal isoform expression (right-sided heatmaps). Tissues are grouped and shaded according to the key at the bottom of the figure. The number of samples per tissue type can be found in Table S1.

We quantified C-terminal isoform expression (resulting from alterative splicing) for *TP73* and *TP63* by analyzing the number of RNA-seq reads spanning exon-exon junctions from exons 10–14 (Fig. 2A). In all tissues except skeletal muscle, p63α was the predominant (67–97%) isoform expressed (Fig. 2B and Table S1). p63β was the second most expressed isoform (5–21%) in epithelial tissues with the highest expression of *TP63* (>10 TPM) (Fig. 2B and Table S1). Consistent with a previously reported study [43], skeletal muscle expressed a high percentage (85%) of p63γ (Fig. 2B and Table S1). p63γ was expressed at much lower relative amounts (0–24%) in all other tissues, and p63δ was expressed at nominal levels in all tissues examined (Fig. 2B and Table S1). Two types of junction-spanning reads were identified between exons 8–9 of *TP63* [53]. Across tissues, we found that full-length E8 was the predominant *TP63* isoform expressed (~70%) and the shortened variant of exon 8 (E8s) was expressed at lower levels (~30%) (Fig. 2A and Table S2).

p73 exhibited greater diversity in C-terminal splicing compared to p63 (Fig. 2B and Table S1). p73α was the predominant C-terminal splicing pattern across most tissues (75–94%) (Fig. 2B and Table S1). Among these same tissues, p73β was the second-highest expressed (4–31%) isoform (Fig. 2B and Table S1). We analyzed p73γ and p73ε in tandem (denoted as y+ε in Fig. 2B and Table S1) because these isoforms have an exon structure that is difficult to differentiate. Together, the p73γ and p73ε C-terminal splicing isoforms were the third most expressed isoform in the majority of tissues studied (0–28%), with the highest percentages of expression found in neuronal tissues from the p73-Low/p63-Low group (Fig. 2B and Table S1). Negligible expression (1–3%) of p73δ and p73ζ was detected in a minority of tissues (Fig. 2B and Table S1).

### Analysis of p73 and p63 N-terminal mRNA expression in human tissue

We anticipated that quantification of N-terminal isoform expression (resulting from differential promoter usage [P1-TA; P2-∆N]) of p73 and p63 (Fig. 2A) would be straightforward similar to C-terminal expression. We analyzed *TP63* splice junction counts from exon 3 (E3) to exon 4 (E4) and exon 3′ (E3′) to E4 to determine canonical promoter usage by tissue. ∆Np63 was the predominant isoform (80–100%) in most tissues from the p73-High/p63-High group (Fig. 1A), including the skin, esophageal mucosa, vagina, and prostate (Fig. S4B and Table S4). The testis was an exception and predominately expressed (96%) TAp63 (Fig. S4B and Table S4). Among the tissues from the p63^High^ group (Fig. 1A), the bladder expressed ∆Np63 (100%), and skeletal muscle expressed TAp63 (100%) (Fig. S4B and Table S4).

**Fig. 3:**
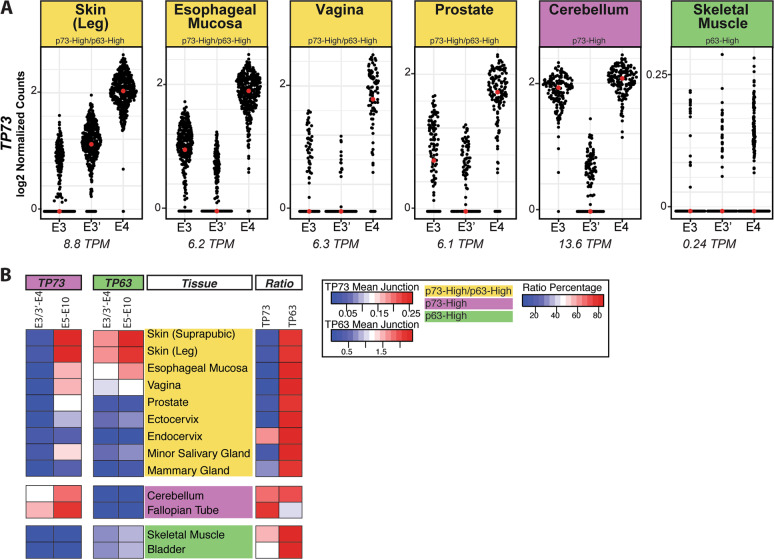
*TP73* N-terminal Expression in Human Tissue. **A** Sina plots of *TP73* N-terminal exon counts for select human tissue types from the GTEx dataset. Exon counts are normalized by exon length and sequencing depth and plotted on a log_2_ scale (normalized counts + 0.01). Each red dot indicates the median value of the population. Tissues are grouped and shaded by the following: p73-High/p63-High (yellow), p73-High (purple), and p63-High (green). The average *TP73* gene expression (units = TPM) of each GTEx tissue is listed below its respective plot. **B** Heatmaps (left-sided) of the mean number of exon junction-spanning reads connecting exon 3 or exon 3′ to exon 4 (E3/3′-E4) and the mean number of junction-spanning reads between exons 5–10 (E5–E10) for both *TP73* and *TP63*. Tissues are grouped and shaded as described in Part A. The right-sided heatmap shows the ratio of E3/E3′–E4 to E5–E10 junction-spanning reads for *TP73* and *TP63*.

While analyzing the expression of individual p73 exons, we observed that the expression level of E4, which is shared by both TA and ∆N p73 isoforms, was disproportionally greater than the combined expression of E3 and E3′ in several epithelial tissues from the p73-High/p63-High group (skin, esophageal mucosa, vagina, and prostate) (Fig. 3A and Table S3). We did not observe a similar difference in the expression levels of E4 versus E3 plus E3′ for *TP63* in the same epithelial tissues (Fig. S4A) or either gene in non-epithelial tissues (skeletal muscle and cerebellum) with either high *TP73* or *TP63* expression (Figs. 3A and S4A, and Table S3). Decreased 5′ RNA-seq coverage can be caused by low-quality or degraded RNA during library preparation, resulting in an inability to generate full-length cDNA during oligo(dT) priming. However, we did not find evidence that samples from epithelial tissues with reduced *TP73* E3 + E3′ versus E4 expression had lower quality RNA as assessed by RNA integrity number (RIN) or post-mortem interval (time from death to tissue collection) [54, 55]. Also, we analyzed the genomic sequence of *TP73* E3′. We determined that it had similar “mappability scores” (a measure of the ability to align short-reads to a sequence) as other exons in the gene (data not shown).

To further evaluate the observed discrepancy in *TP73* N-terminal exon expression among epithelial tissues analyzed in Fig. 3A, we turned to isoform junction expression data since it is impacted less by variations in exon length. We calculated the number of exon junction-spanning reads between E3–E4, E3′–E4 and the other exon junctions shared by all canonical p73 isoforms (E5–E10) (Fig. 3B and Table S5). These data were used to calculate the ratio (as a percentage) of the sum of *TP73* E3/3′–E4 expression to the mean junction expression of E5–E10 in each tissue (Fig. 3B and Table S5). The same analysis was performed for *TP63* for comparison (Fig. 3B and Table S5). Consistent with the exon expression from the p73-High/p63-High group (Fig. 3A), *TP73* E3/3′–E4 junctional reads were significantly reduced (~90%) compared to the mean of all junctional reads across E5–E10 (Fig. 3B and Table S5). However, in p73-High tissues (cerebellum and fallopian tube), *TP73* E3/3′–E4 junctional expression was only reduced by ~30% compared to E5–E10 (Fig. 3B and Table S5). The calculated ratio percentage for *TP73* in the testis is not accurate because the tissue utilizes a unique promoter (I3a) to express an mRNA lacking exons 5–10; thus, we excluded it from the analysis. In line with our prior findings (Fig. 3A), *TP63* E3/3′–E4 junction expression was ~75% of the mean of junctions E5–E10 in tissues from the p73-High/p63-High group (Fig. 3B and Table S5), an amount consistent with the expected degree of 3′ bias typically seen in high-quality RNA-seq experiments utilizing poly-A capture. In sum, these findings suggest that the decrease of *TP73* E3/3′–E4 junction expression is specific to epithelial tissue from the p73-High/p63-High group, and not merely due to tissue-specific characteristics since a similar reduction in E3/3′–E4 junction expression was not observed for *TP63* in these same tissues.

### Analysis of *TP73* transcription start site usage in human epithelial tissue

Having identified select epithelial tissues with reduced expression of *TP73* E3 and E3′, we considered different possibilities for the discordant expression pattern. One such explanation is alternative splicing between exons 1 (E1) or 2 (E2) and E4. GTEx did not report any junctional reads between these exons, and manual review of individual alignments did not find evidence of E1–E4 or E2–E4 junction-spanning reads (data not shown). Another possibility is the existence of an additional transcriptional start site (TSS) in the *TP73* locus located at or upstream of E4. Such a transcript would contain an in-frame methionine at the beginning of E4, which could encode for a protein similar in size to ∆Np73. To investigate this possibility, we used publicly available 5′-end RNA-seq data for GTEx samples produced by the ENCODE Project [56, 57]. These data were generated using the RAMPAGE (RNA Annotation and Mapping of Promoters for the Analysis of Gene Expression) approach, an RNA-seq method to identify TSS at single-base resolution with a high signal-to-noise ratio across the genome [56].

**Fig. 4:**
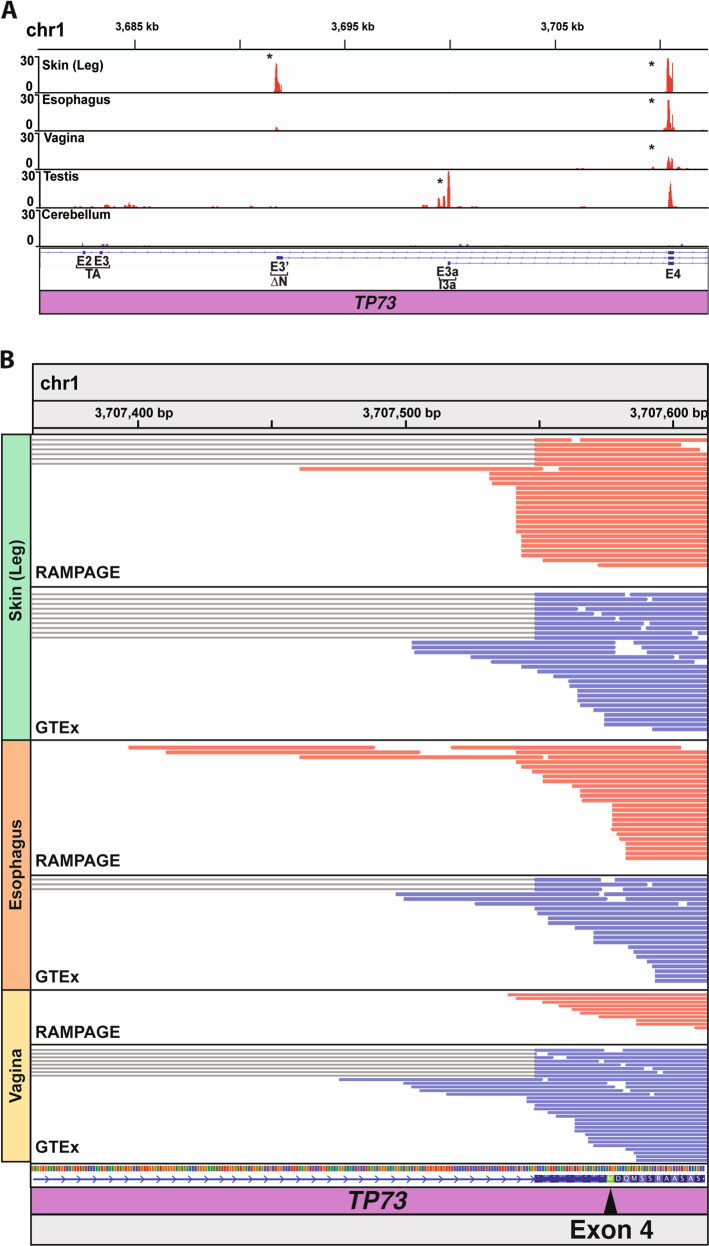
Differential *TP73* TSS usage in human tissue. **A** Genome browser view of the 5′ end of the *TP73* genomic locus showing the read density of 5′-end RNA-seq (RAMPAGE) for select human tissue from the ENCODE Project. RAMPAGE peaks (i.e., TSS) were identified using the ENCODE standard RAMPAGE pipeline and are marked with an asterisk. **B** Genome browser view of *TP73* exon 4 showing aligned reads from 5′-end (RAMPAGE, red-colored reads) and poly-A (GTEx, blue-colored reads) RNA-seq performed on the same skin, esophagus, and vagina samples. The thin gray lines annotate reads aligning to the junctions between exon 3 or 3′ and exon 4. The methionine within exon 4 (green colored, marked with an arrowhead) is the putative start codon of E4p73.

We observed a RAMPAGE peak immediately upstream of *TP73* E4 in multiple epithelial tissues from the p73-High/p63-High group, including skin (leg), esophagus, and vagina (Fig. 4A), that would align with an in-frame methionine in E4 (Fig. 4B, arrowhead). Testis also had RAMPAGE read coverage at this peak, but it did not reach genome-wide significance (Fig. 4A). In contrast, the cerebellum, which did not exhibit decreased relative expression of *TP73* E3 and E3′ (Fig. 3B), lacked read coverage at the peak (Fig. 4A). A RAMPAGE peak was found at the canonical ∆Np73 TSS (5′ end of E3′) in only skin (Fig. 4A). The esophagus and testis also had RAMPAGE reads at this peak that did not achieve genome-wide significance (Fig. 4A). We detected a RAMPAGE peak at the 5′ end of *TP73* exon 3a (E3a), specifically in testis (Fig. 4A), the only tissue that expresses significant levels of a transcript composed of only E3a and E4 (data not shown). None of the tissues we investigated had a RAMPAGE peak at the canonical TAp73 TSS at E1 (data not shown). As a control, we analyzed the RAMPAGE peaks of *TP63* in the same samples. The only peak detected in *TP63* was located at the canonical ∆Np63 TSS (5′ end of E3′) and found in skin, esophagus, and vagina (Fig. S5A).

To further assess the potential for a TSS upstream of *TP73* E4, we analyzed the RAMPAGE 5′-end, and RNA-seq reads aligning to this region. In each epithelial tissue with a *TP73* E4 peak (Fig. 4A), we found canonical E3–E4 junction-spanning reads in addition to several non-overlapping reads aligning to the intronic region immediately upstream of E4 (Fig. 4B). The maximum length of RNA-seq read coverage upstream of the early methionine in E4 (i.e., putative UTR) was 115 bp for skin, 179 bp for esophagus, and 100 bp for vagina (Fig. 4B). We also analyzed the *TP73* read alignments to E3′ (Fig. S6A) and confirmed they were consistent with its known role as a UTR and our previous analyses (Figs. 3A and 4A). In addition, we analyzed *TP73* E7 (Fig. S6B), an exon shared by all isoforms, and ruled out the presence of reads aligning to intronic regions (which can be seen when samples for RNA-seq are contaminated with genomic DNA or pre-mRNA). Lastly, as a control, we performed a corresponding analysis of *TP63* E3′ and E4 read alignments and did not find any reads aligning to the intronic region upstream of *TP63* E4 (Figs. S5 and S7). Collectively, these results provide initial evidence for the existence of a previously unreported (to our knowledge) *TP73* mRNA product transcribed in select epithelial tissue from a TSS immediately upstream of E4.

### Analysis of *Tp73* and *Tp63* mRNA in murine tissues

To extend our p63 and p73 isoform findings to another organism, and analyze primary tissue instead of in silico data sets, we designed appropriate primers as described in “Materials and methods” section and performed qRT-PCR and IF. We analyzed p63 isoform expression in murine skin, vagina, mammary gland, and muscle tissue by qRT-PCR (Fig. S8A). Further, we performed p63 IF on murine vagina and skin tissue (Figs. S2 and S3) and observed congruent protein expression with vagina having the highest level.

Murine TAp73 and ∆Np73 mRNA expression patterns were similar to those observed in human GTEX data (Fig. S8B). Using maximum shared upstream read length from human tissues as a guide (~100 bp), we designed murine qRT-PCR primers to evaluate E4p73. We observed expression of a mRNA product corresponding to E4p73 in the murine tissues investigated (Fig. S8B). p73 IF analyses showed that protein expression in murine skin, vagina, mammary gland, and muscle tissue correlates highly with mRNA expression (Figs. S2 and S3).

In summary, p73 and p63 expression are positively correlated across a wide range of normal human and murine tissues. p73 is highly expressed in a subset of p63-positive cells within the epithelial lining of the tissues in which they are co-expressed. As previously reported, p63 is predominately expressed as the ∆Np63α isoform, while p73 isoform expression is more varied. We have also identified a potential new TSS of p73 that could lead to a protein product that initiates from a methionine in E4.

## Discussion

The analysis of p73 and p63 has been challenging due to the lack of isoform-specific antibodies and the large number of isoforms that can be generated from each gene. The development of high-throughput sequencing technologies and their application to transcriptome profiling has made it possible to quantitatively survey the entire transcriptome at single-base resolution [58]. Our analysis of the *TP73* and *TP63* gene-level expression data from GTEx validated previously published tissue-specific gene expression analyses [5, 16, 28, 59–61]. In addition, the expression patterns of *TP73* and *TP63* in human tissues, reported herein, aligns with the murine tissues in which phenotypes have been reported using p73-deficient and p63-deficient mice [17, 19, 20, 31, 35, 36, 38, 41, 45, 48–50, 60, 62].

We were motivated to study the protein expression pattern of p73 and p63 because of the physical and functional interactions between the two transcription factors, and the lack of a comprehensive atlas of their co-expression and localization across tissues. Given the known roles of both p73 and p63 in stem cell regulation, the high co-expression of both proteins in the basal layer of epithelial tissues (e.g., skin, esophagus, vagina, endocervix, and ectocervix) is of interest. Despite the lack of an overt skin phenotype in p73 knockout mice, we recently found that p73 is important in the process of timely epidermal wound healing [19]. Thus, we hypothesize that additional phenotypes would be observed in barrier epithelial tissues with high levels of p73 expression after damage.

Our study has provided initial evidence supporting the existence of a previously unreported p73 mRNA product in multiple epithelial tissues beginning in the intron upstream of E4. A previous study confirmed that the E4 start codon in ΔNp73α could initiate translation and produce a ∆Np73-like protein product using in vitro translation and cell-based overexpression studies [63]. Further, studies in small cell lung cancer and ovarian carcinoma have observed increased translation initiation from the methionine in E4 in connection to alternative splicing of TAp73, ∆ex2p73, and ∆ex2/3p73 [64, 65]. We did not observe splicing of E4 to either E2 or E1 in the GTEx data; rather, we observed a unique TSS in the intronic region directly upstream of E4, potentially generating a putative UTR upstream of an early E4 methionine. E4 initiation was previously reported for p63 when a truncated ∆Np63 protein was produced by translation re-initiation at the first methionine in E4 in three patients with an ankyloblepharon-ectodermal dysplasia-cleft lip/palate-like syndrome [66]. It will be important to determine the relative expression level and biological differences between ∆Np73 and E4p73 in physiologically-relevant contexts. The first 13 amino acids of ∆Np73 isoforms are important in transcriptional activation of downstream target genes [9]. Since E4p73 lacks the first 22 amino acids shared in ∆Np73, we predict that E4p73 would have reduced transcriptional activity compared to ∆Np73 due to the absence of the novel activation domain [9]. Further work is also needed to determine how E4p73 alters *TP73* function since most tissues express multiple N-terminal isoforms.

To our knowledge, this study provides the most extensive bioinformatic analysis of *TP73* and *TP63* mRNA isoform expression across human tissues to date coupled with an atlas of protein expression patterns at the cellular level in humans and mice. Given the rigorous procedures for tissue procurement and RNA-seq used by the GTEx Project [67], the analyses herein should be useful to investigators interested in tissue-specific expression and the functional relevance of the various p73 and p63 isoforms. The results presented herein provide further evidence that the study of p73 and p63 in isolation offers only a partial view of potential signaling outputs, as the proteins are co-expressed in a wide array of cell types and can heterodimerize. Future studies should investigate both proteins in concert to understand their independent and dependent functions better.

## Materials and methods

### Human and murine sample acquisition

Human tissue sections were selected by a board-certified pathologist (M.E.S) under the auspices of IRB # 192076, “Normal Tissue Atlas.” Murine studies described herein were carried out according to recommendations in the Guide for the Care, and Use of Laboratory Animals of the National Institutes of Health (NIH), and protocols used were approved by the Institutional Animal Care and Use Committee (IACUC) of Vanderbilt University Medical Center (VUMC) (Protocol Number: M1800069). All IF staining of human tissue was conducted in triplicate on samples from at least three different individuals. Murine IF experiments were conducted in male and female mice from BALB/c and C57BL/6 backgrounds in triplicate to ensure staining results were not biased by gender or strain.

### Immunofluorescence (IF)

Immunostaining of tissue sections was performed as previously described [17]. IF was conducted using the antibodies p73 EP436Y (Abcam ab40658; 1:1000; Cambridge, UK) and p63α D2K8X (Cell Signaling Technology #13109; 1:1000; Danvers, MA), with TSA Plus Fluorescence Amplification Kit (Perkin Elmer, Waltham MA). Nuclei were counterstained with Slowfade Gold DAPI mounting media (Invitrogen, Carlsbad, CA). Micrographs of human tissues were all taken using a Zeiss slide scanner and further analyzed using Zeiss software. Micrographs of murine tissues were taken using a Leica microscope and camera and analyzed using Leica software; all scale bars = 50 µm.

### Genotype-tissue expression (GTEx) RNA-seq analysis

RNA-seq data for 11,688 human samples (51 normal tissue sites and two primary cell lines) was downloaded from the GTEx Portal on January 1, 2018 (V7 Release) [67, 68]. The RNA-seq libraries were generated from total RNA using the Illumina TruSeq RNA Library Preparation Kit (non-stranded, poly-A capture) and sequenced on the Illumina HiSeq 2000 or 2500 (>50 million 76 base pair paired-end reads per sample). Sequencing reads were aligned to hg19 with STAR v2.4.2a [69] using GENCODE v19 [70] annotations. Gene-level expression (units = TPM) were quantified using RNA-SeQC v1.1.8 [71] and junction read counts using STAR v2.4.2a [69]. For each sample, the number of reads spanning each *TP73* and *TP63* exon–exon junction was calculated, normalized to the counts per million (read counts/total number of aligned reads/1e6), and used to determine isoform expression for the N-terminus and C-terminus independently. The relative amount of normalized junction counts for exon 3 to exon 4 (E3–E4; corresponds to the TA isoform) versus exon 3′ to exon 4 (E3–E4; corresponds to the ∆N isoform) was used to determine the percentage of N-terminal isoform expression for both genes. For *TP73*, the relative amount of exon 3a to exon 4 (E3a–E4; corresponds to the I3a isoform) was also incorporated into the analysis. The GTEx junction count quantification data set did not contain information on the exon-spanning junctions corresponding to three previously identified N-terminal isoforms (Ex2p73, Ex2/3p73, and ∆Np73′) [72]. Junction counts for *TP63* corresponding to exon 4 skipping (exon 3 or exon 3′ to exon 5) and an additional 216 base pair exon (located in the intron between exons 4 and 5) were detected at very low levels and thus excluded from the analysis (lacked tissue-specific expression, their expression simply correlated with total *TP63* expression). C-terminal isoform expression for both genes was calculated similarly with slight modifications for each gene to account for differences in alternative splicing between them. The percentage of p73α + β [exon 10 to exon 11 (E10–E11)], p73γ + ε [exon 10 to exon 12 (E10–E12)], p73ζ [exon 10 to exon 13 (E10-E13)], and p73δ [exon 10 to exon 14 (E10–E14)] was calculated by analyzing normalized exon–exon junction counts starting at exon 10 (E10). The relative expression of p73α versus β was determined by comparing normalized exon 12 to exon 13 (E12–E13; corresponds to the α isoform) versus exon 12 to exon 14 (E12–E14; corresponds to the β isoform) junction counts. The percentage of p63γ [exon 10 to exon 11a (E10–E11a)] versus non-p63γ (E10–E11) was calculated by analyzing normalized exon–exon junction counts starting at E10. The percentage of p63δ [exon 11 to exon 14 (E11–E14)] versus non-p63δ (E11–E12) expression was calculated, including normalization for the previously determined percentage of p63γ expression (multiplied relative percentages of p63δ and non-p63δ by the non-p63γ from the prior calculation). The percentage of p63β (E12–E14) and p63α (E12–E13) was calculated using the same methodology as p63δ. Isoform expression for each tissue was calculated as the mean isoform expression for all samples belonging to a given tissue. We calculated the ratio (as a percentage) of the E3/3′-E4 junction expression to mean junction expression of E5–E10 for *TP73* and *TP63* in each tissue using the following equation: (sum of E3/3′–E4 normalized expression)/(mean normalized expression of exon junctions between E5–E10) × 100. A total of 36 tissues were included in the final results (Table S1). The GTEx ID’s of individual samples we show in Fig. 4 were Skin (Leg) (GTEX-1GF9X-1326-SM-7PC2Y), Esophagus (GTEX-1LGRB-1626-SM-CNNR7) and Vagina (GTEX-1HCUA-2426-SM-ADEIS).

### Encyclopedia of DNA elements (ENCODE) TSS 5′-end RNA-seq analysis

RAMPAGE (RNA Annotation and Mapping of Promoters for the Analysis of Gene Expression) [56, 57] data for human adult and embryonic tissues with TP73, and/or TP63 expression (*n* = 17) was downloaded from the ENCODE Project Portal [73] on December 8, 2019. TSS identified by the ENCODE RAMPAGE pipeline were manually reviewed in IGV [74, 75] for each sample to validate that the signal for unique reads at the *TP73* and *TP63* genomic loci was consistent with TSS calls. The ENCODE ID’s of individual samples shown in Fig. 4 are: Skin (Leg) (ENCFF174CGG), Esophagus (ENCFF970OJS), Vagina (ENCFF838AHK), Prostate (ENCFF362JKL), and Cerebellum (ENCFF569IAA).

### Murine RNA preparation and qRT-PCR analysis

RNA was harvested from tissue by homogenizing murine muscle, mammary, vagina, and dorsal skin samples in Trizol and purified using the Aurum Total RNA Mini kit (Bio-Rad) in triplicate. qRT-PCR experiments were conducted using oligo(dT)-mediated first-strand synthesis and SYBR Green quantification. mRNA levels were quantified using primers targeting **TAp73** (exon 3–4 junction) (TAp73 Forward: 5′ GTTGGGGAGATGGCCCAGACCT 3′/TAp73 Reverse: 5′ CCATGTTGGACTCCTCGCTGCC 3′), **∆Np73** (exon 3′–4 junction) (∆Np73 Forward: 5′ CACACCAGCTCCTCAGCGTGTG 3′/∆Np73 Reverse: 5′ CTGGTCCATGGCACTGCTGAGC 3′) **E4p73** (Exon4) (E4p73 Forward: 5′ TGTTTCTCCCCTCCCACCTCCC 3′ /E4p73 Reverse: 5′ CCCATCTGGTCCATGGCACTGC 3′) and **TAp63** (exon 3–4 junction) (TAp63 Forward: 5′ TGCCACCCTACAGTACTGCCCC 3′/TAp63 Reverse: 5′ CTCGCTTGTCTGGGTGCTCTGC 3′), **∆Np63** (exon 3′–4 junction) (∆Np63 Forward: 5′ GCAGCCTTGACCAGTCTCACTGC 3′/∆Np63 Reverse: 5′ TCCATGCTGTTCAGGAGCCCCA 3′). All of the isoform specific qRT-PCR levels were normalized to the expression level of GAPDH using the primers (GGGGTCGTTGATGGCAACA/ AGGTCGGTGTGAACGGATTTG).

### Statistical analysis

All statistical analyses and graphical representations were conducted using R (version 3.5.2 or 3.5.3) unless otherwise noted. The Spearman correlation between *TP63* and *TP73* expression across human tissues (GTEx RNA-seq dataset), and its statistical significance were calculated using the “cor.test” function in R.

## Supplementary information

Table S1

Table S2

Table S3

Table S4

Table S5

Supplemental Figure Legends

Figure S1

Figure S2

Figure S3

Figure S4

Figure S5

Figure S6

Figure S7

Figure S8

## Data Availability

All code used for the analysis of GTEx data can be found on Figshare (10.6084/m9.figshare.14684190). File “20210526-GTEx_data_processing.sh” contains the code used to process GTEx RNA-seq data in order to obtain mRNA expression data for *TP73* and *TP63*. The file “20210526-key_computational_analyses.R” contains the code used to quantify and graph *TP73* and *TP63* mRNA expression at the gene, exon, and isoform level.
