# Supplementary material for: Tissue-specific expression of p73 and p63 isoforms in human tissues
Source: Cell Death Dis. 2021 Jul 27;12(8):745. doi: 10.1038/s41419-021-04017-8 (PMC8316356; doi:10.1038/s41419-021-04017-8)
Supplement: Supplementary file 6 — Supplemental Figure Legends [file 41419_2021_4017_MOESM6_ESM.docx]

**SUPPLEMENTAL FIGURE LEGENDS**

**Figure S1. p73 and p63 Protein Expression in Human Tissue.** Representative dual IF micrographs of p73 (red), p63 (green), and nuclei (blue) alongside H&E stained serial sections from human: (A) p63-High tissues (skeletal muscle and bladder), (B) p73-High tissues (cerebellum and fallopian tube), and (C) p73-High/p63-High skin tissue (taken from the face, abdomen/groin, and gluteus). For each normal tissue site, samples from three different humans were stained and analyzed to identify the representative area shown. Scale bars = 50µm.

**Figure S2. p73 and p63 Protein Expression in Murine Tissue.** Representative dual IF micrographs of p73 (red), p63 (green), and nuclei (blue) from murine: (A) p63-High tissues (skeletal muscle and bladder), (B) p73-High tissues (cerebellum), and (C) p73-High/p63-High tissues (esophagus, vagina, salivary gland, mammary and prostate). For each normal tissue site, samples from three different BalbC and C57B6 (mixed genders) mice were stained and analyzed to identify the representative micrographs shown. Scale bars = 50µm.

**Figure S3. p73 and p63 Protein Expression in Murine Skin Tissue.** Representative dual IF micrographs of p73 (red), p63 (green), and nuclei (blue) from murine skin tissue (dorsal back, toe, ear, and tongue). For each normal tissue site, samples from three different BalbC and C57B6 (mixed genders) mice were stained and analyzed to identify the representative micrographs shown. Scale bars = 50µm.

**Figure S4. *TP63* N-terminal Expression in Human Tissue** (A) Sina plots of *TP63* N-terminal exon counts for select human tissue types from the GTEx dataset. Exon counts are normalized by exon length and sequencing depth and plotted on a log_2_ scale (normalized counts + 0.01). Each red dot indicates the median value of the population. Tissues are grouped and shaded by the following: p73-High/p63-High (yellow), p73-High (purple), and p63-High (green). The average *TP63* gene expression (units = TPM) of each GTEx tissue is listed below its respective plot. (B) Heatmap of *TP63* N-terminal isoform expression calculated through analysis of exon-exon junction-spanning reads. The mean percentage expression of TA and ∆N isoforms for each tissue type can be found in Table S4.

**Figure S5.** ***TP63* TSS Usage in Human Tissue**. (A) Genome browser view of the 5’ end of the *TP63* genomic locus showing the read density of 5'-end RNA-seq (RAMPAGE) for the same human tissue shown in Figure 4A (from the ENCODE Project). RAMPAGE peaks (i.e., TSS) were identified using the ENCODE standard RAMPAGE pipeline and are marked with an asterisk. (B) Genome browser view of *TP63* exon 3' showing aligned reads from 5’-end (RAMPAGE, red-colored reads) and poly-A (GTEx, blue-colored reads) RNA-seq performed on the same skin, esophagus, and vagina samples as Figure 4B. The thin gray lines annotate reads aligning to the junctions between exon 3 or 3’ and exon 4. The methionine within exon 3' (green colored, marked with an arrowhead) is the start codon of ∆Np63.

**Figure S6. *TP73* Transcript Expression at Exon 3’ and 7**. Genome browser view of *TP73* exon 3' (A) and 7 (B) showing aligned reads from 5’-end (RAMPAGE, red-colored reads) and poly-A (GTEx, blue-colored reads) RNA-seq performed on the same skin, esophagus, and vagina samples as Figure 4B. The thin gray lines annotate reads aligning to the junctions between (A) exon 3 or 3’ and exon 4; (B) exon 6 and 7 or exon 7 and 8. The methionine within exon 3' (A; green-colored, marked with an arrowhead) is the start codon of ∆Np73.

**Figure S7.** ***TP63* Transcript Expression at Exon 4.** Genome browser view of *TP63* exon 4 showing aligned reads from 5’-end (RAMPAGE, red-colored reads) and poly-A (GTEx, blue-colored reads) RNA-seq performed on the same skin, esophagus, and vagina samples as Figure 4B. The thin gray lines annotate reads aligning to the junctions between exon 3 or 3’ and exon 4.

**Figure S8. *qRT-PCR of Tp63* and *Tp73* From Murine Tissues**. Schematic representations of exons of *Tp63* (A) and *Tp73* (B) with isoform specific qRT-PCR primers annotated within exons and intron as depicted with blue (TA), red (∆N) and purple (E4p73) lines. mRNA levels of *Tp63* isoforms (A) and *Tp73* isoforms (B) relative to expression of the control GAPDH are graphed with bars representing the mean of triplicate tissue samples as indicated; error bars represent standard deviation.
