# Supplementary figures and images for: Tissue-specific expression of p73 and p63 isoforms in human tissues
Source: Cell Death Dis. 2021 Jul 27;12(8):745. doi: 10.1038/s41419-021-04017-8 (PMC8316356; doi:10.1038/s41419-021-04017-8)

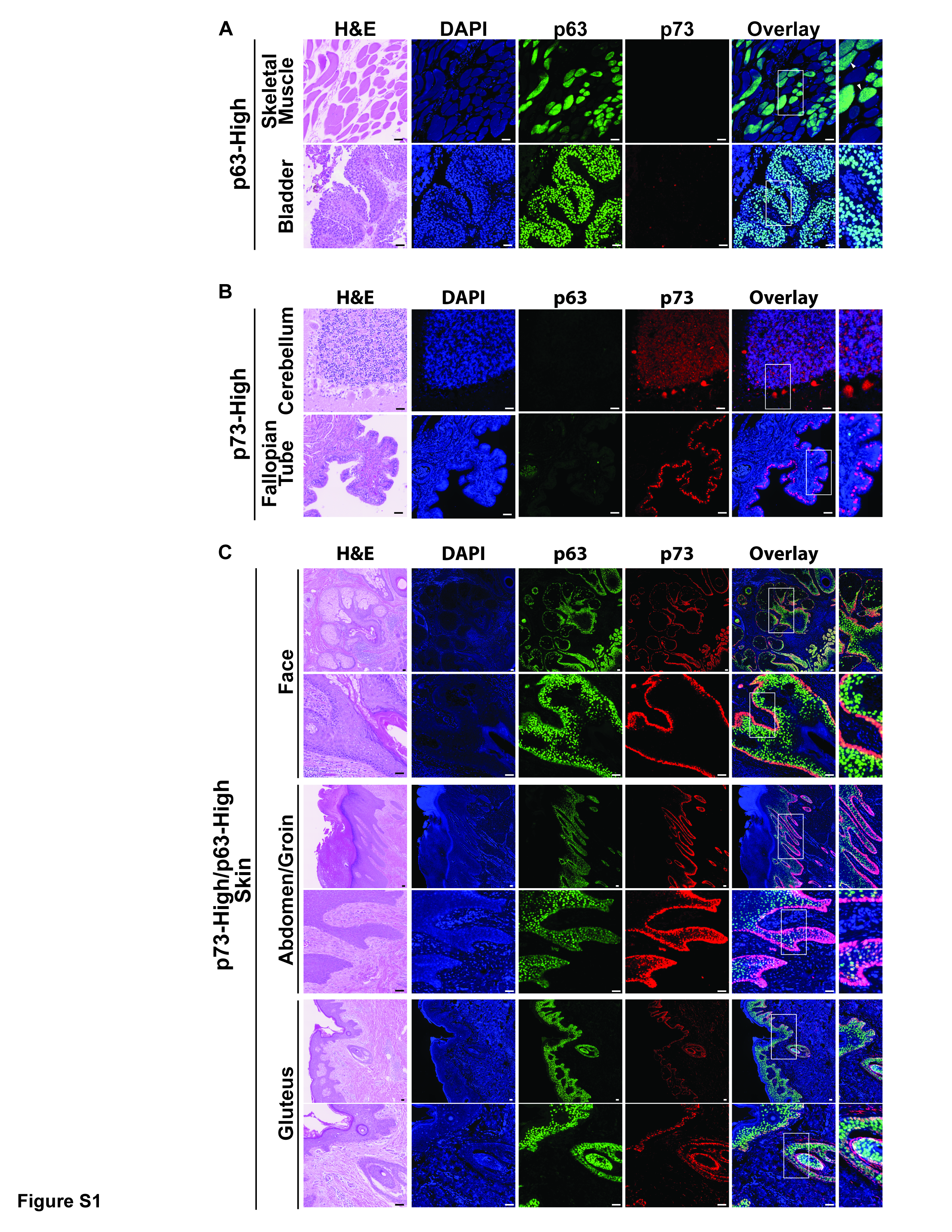

Supplement: Supplementary file 7 — Figure S1 [file 41419_2021_4017_MOESM7_ESM.tif]

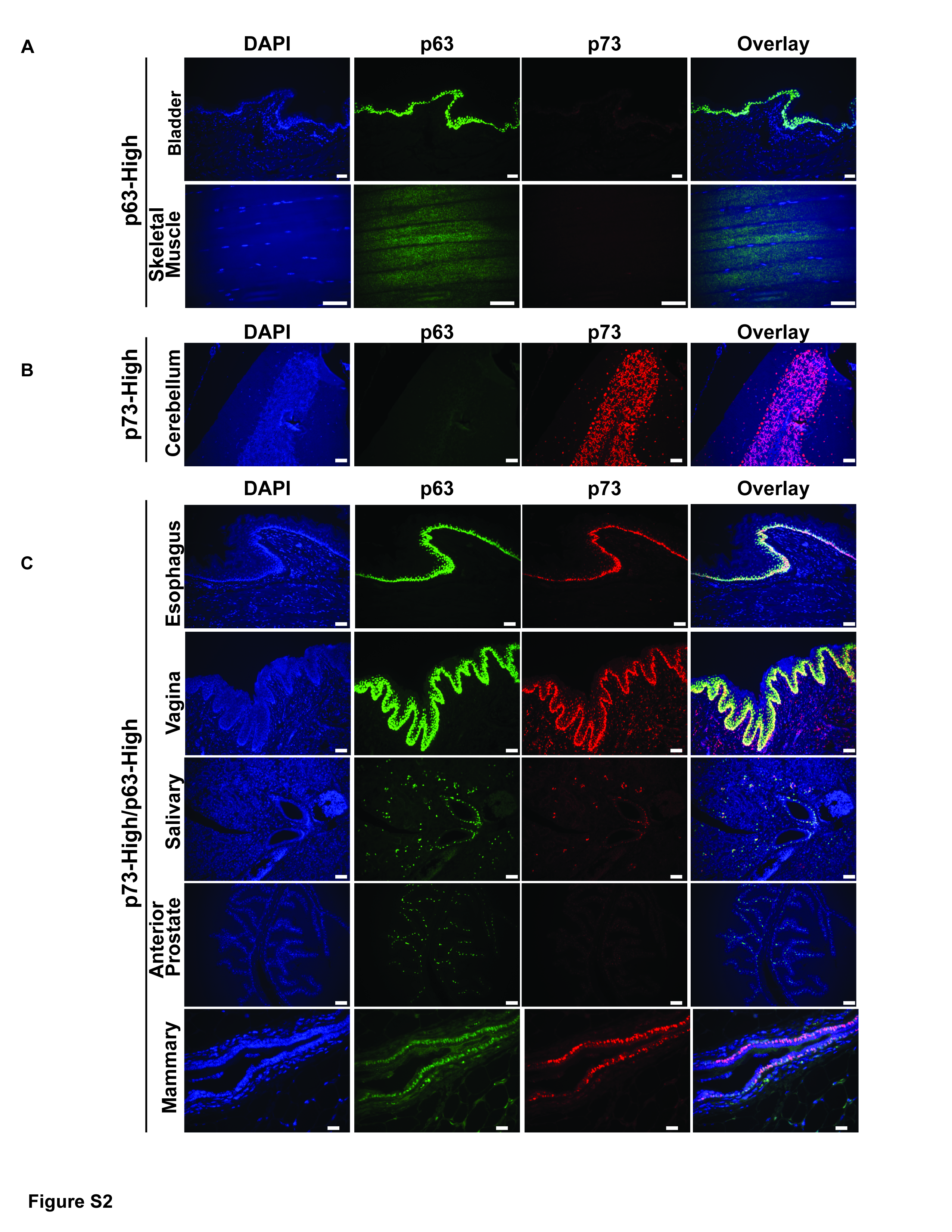

Supplement: Supplementary file 8 — Figure S2 [file 41419_2021_4017_MOESM8_ESM.tif]

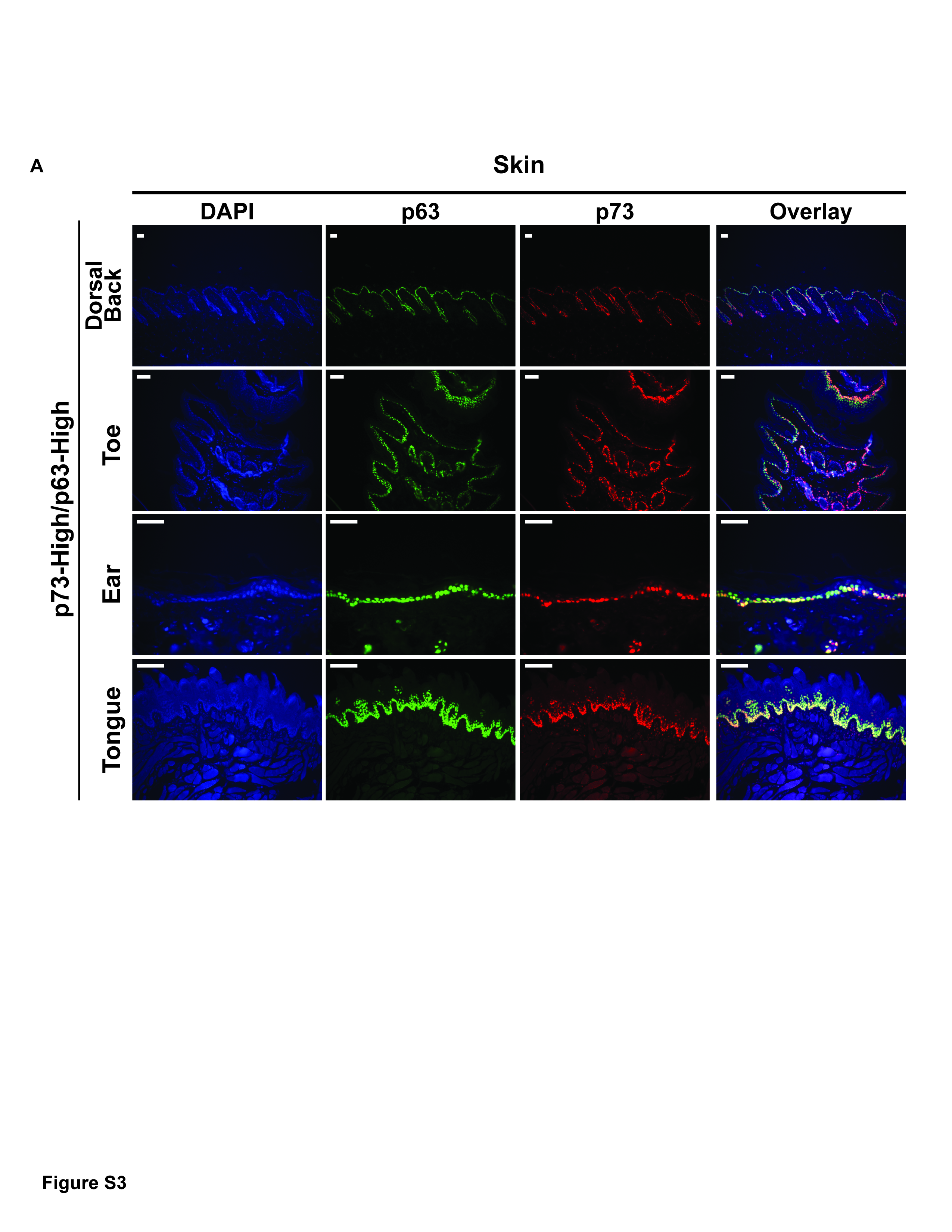

Supplement: Supplementary file 9 — Figure S3 [file 41419_2021_4017_MOESM9_ESM.tif]

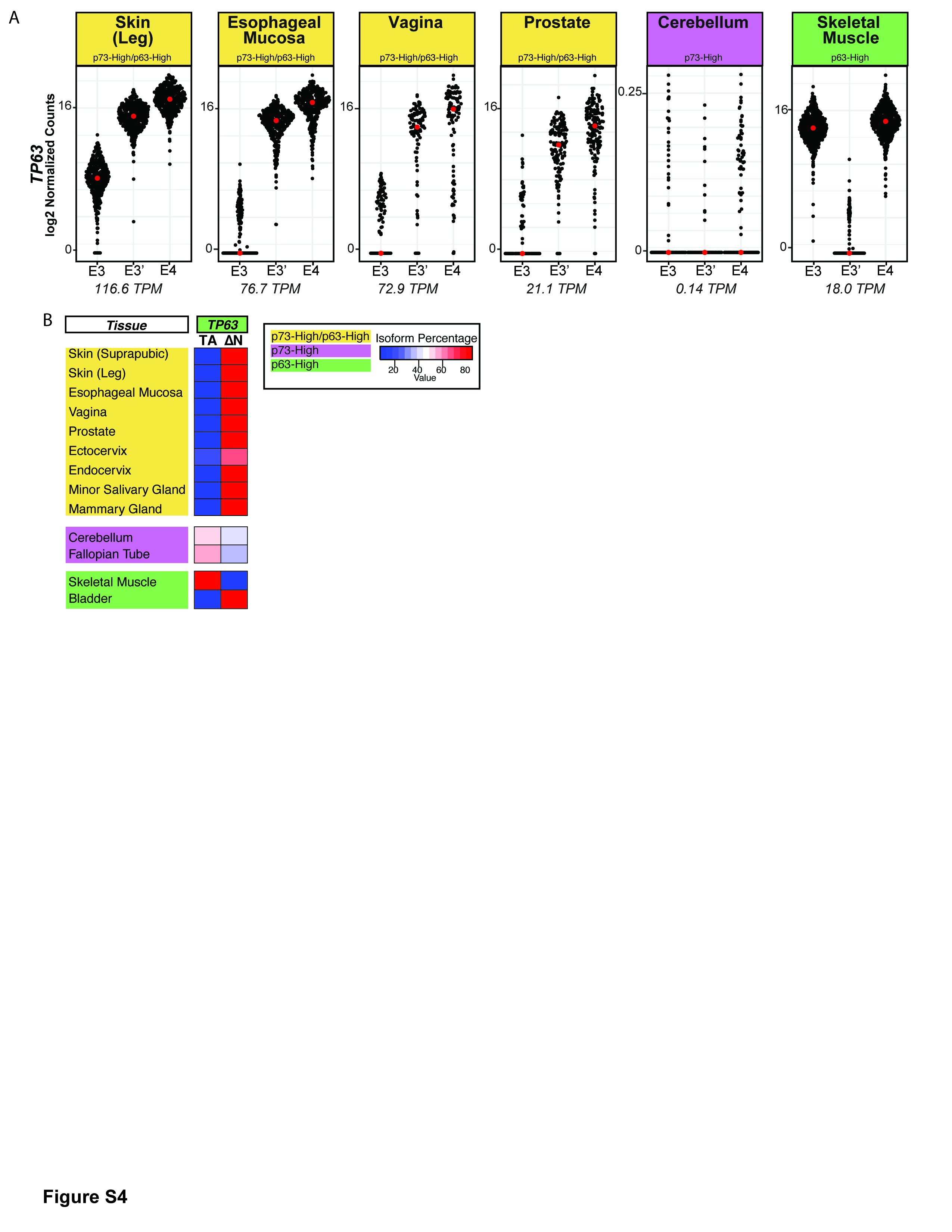

Supplement: Supplementary file 10 — Figure S4 [file 41419_2021_4017_MOESM10_ESM.tif]

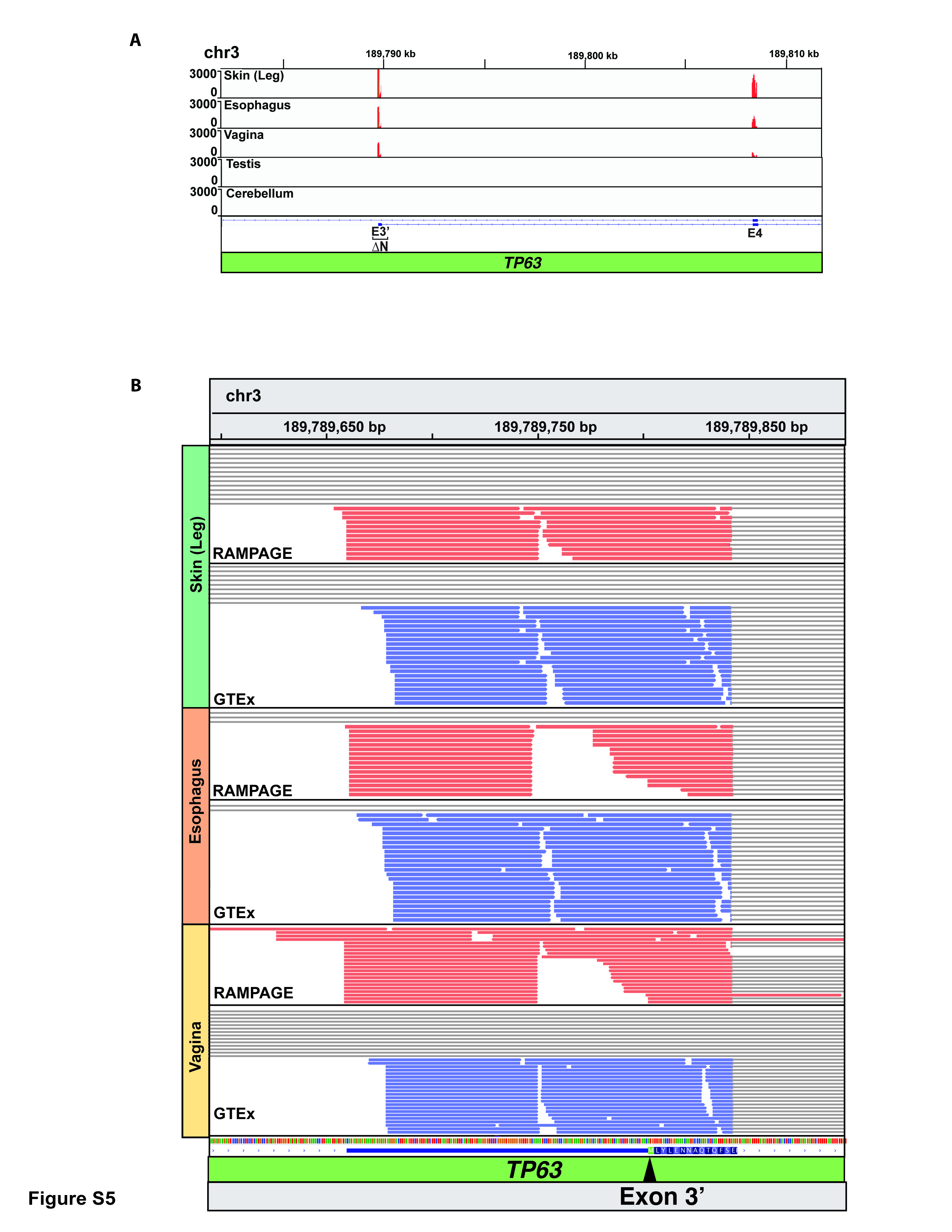

Supplement: Supplementary file 11 — Figure S5 [file 41419_2021_4017_MOESM11_ESM.tif]

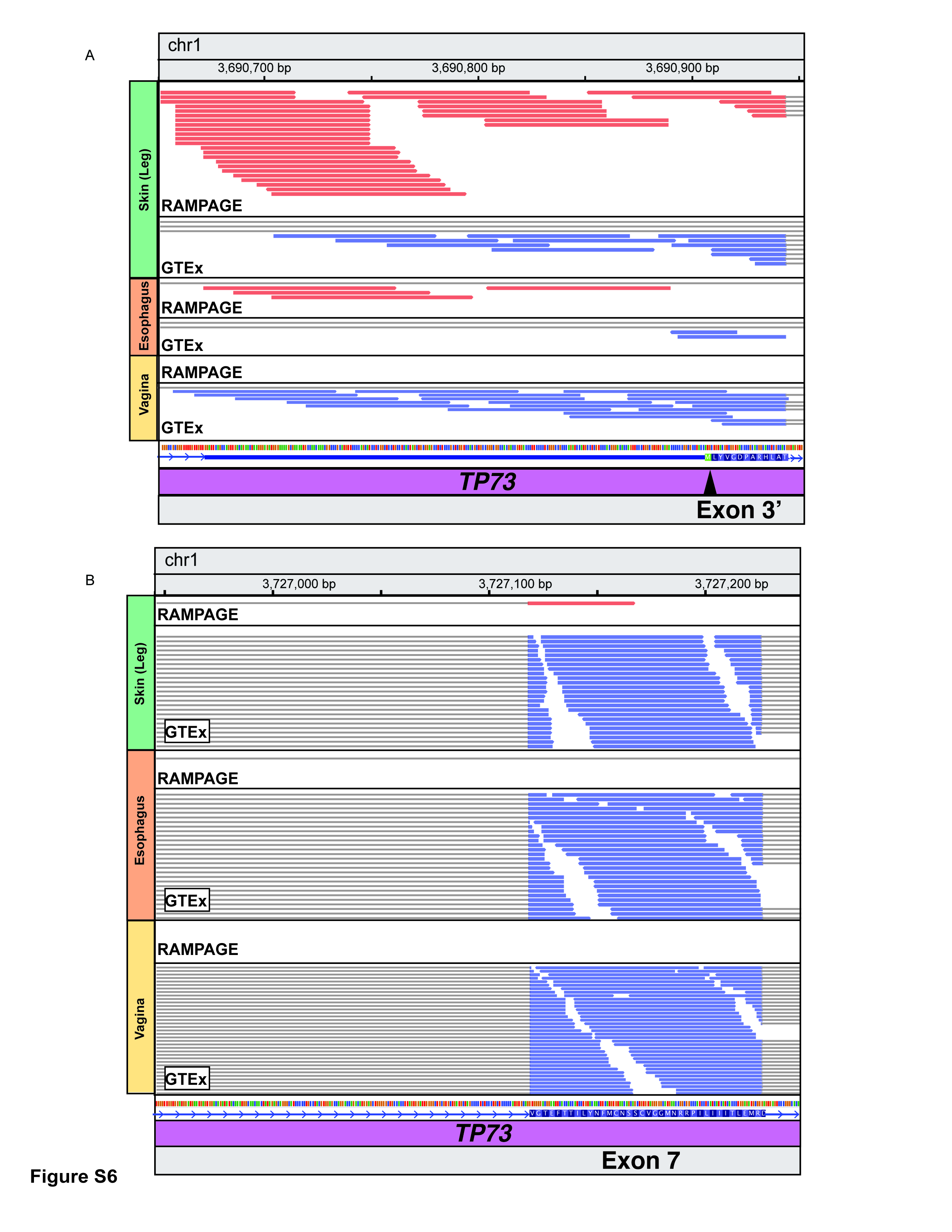

Supplement: Supplementary file 12 — Figure S6 [file 41419_2021_4017_MOESM12_ESM.tif]

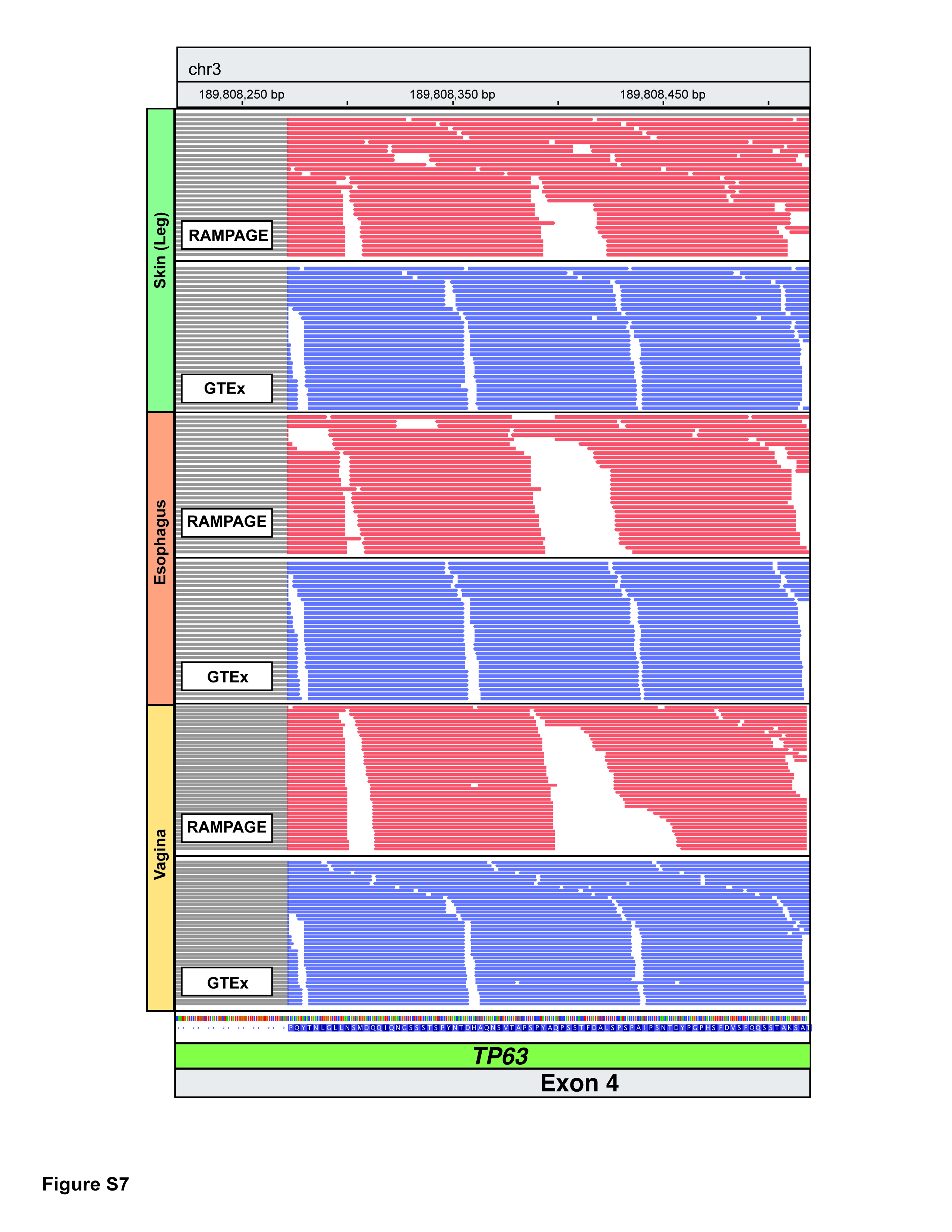

Supplement: Supplementary file 13 — Figure S7 [file 41419_2021_4017_MOESM13_ESM.tif]

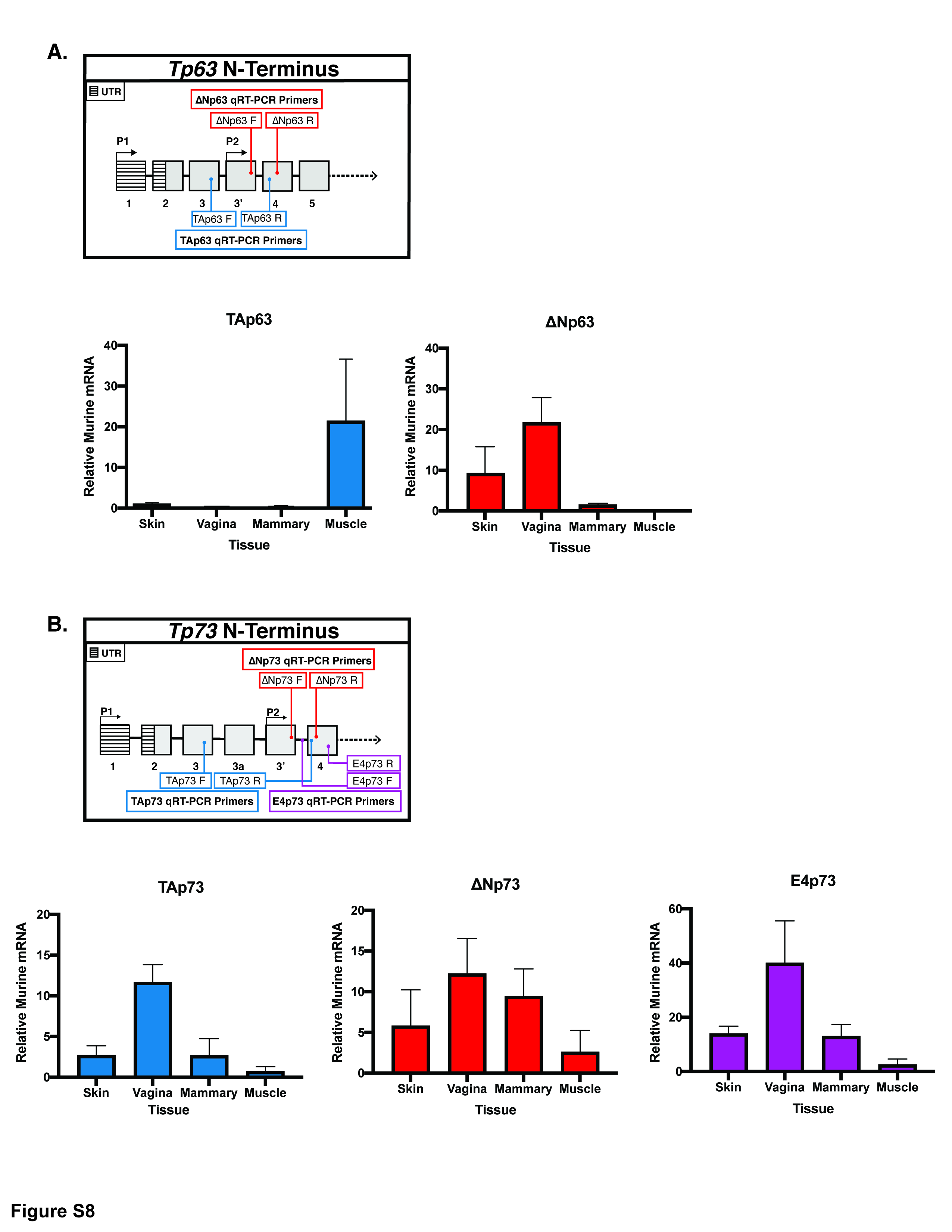

Supplement: Supplementary file 14 — Figure S8 [file 41419_2021_4017_MOESM14_ESM.tif]
